# M2 Cortex Circuitry and Sensory-Induced Behavioral Alterations in Huntington's Disease: Role of Superior Colliculus

**DOI:** 10.1523/JNEUROSCI.1172-22.2023

**Published:** 2023-05-03

**Authors:** Sara Conde-Berriozabal, Lia García-Gilabert, Esther García-García, Laia Sitjà-Roqueta, Xavier López-Gil, Emma Muñoz-Moreno, Mehdi Boutagouga Boudjadja, Guadalupe Soria, Manuel J Rodríguez, Jordi Alberch, Mercè Masana

**Affiliations:** ^1^Department of Biomedical Sciences, Institute of Neurosciences, School of Medicine and Health Sciences, University of Barcelona, 08036, Barcelona, Spain; ^2^Institut d'Investigacions Biomèdiques August Pi i Sunyer, Barcelona, 08036, Spain; ^3^Centro de Investigación Biomédica en Red sobre Enfermedades Neurodegenerativas, Madrid, 28029, Spain; ^4^Magnetic Resonance Imaging Core Facility, Institut d'Investigacions Biomèdiques August Pi i Sunyer, Barcelona, 08036, Spain; ^5^Metofico Ltd, London, WC2H 9JQ, United Kingdom; ^6^Laboratory of Surgical Neuroanatomy, Institute of Neurosciences, School of Medicine and Health Sciences, Universitat de Barcelona, 08036, Barcelona, Spain; ^7^Centro de Investigación Biomédica en Red de Bioingeniería, Biomateriales y Nanomedicina, Madrid, 28029, Spain

**Keywords:** approach/defensive behavior, fiber photometry, mouse fMRI, movement disorders, optogenetics, premotor cortex.

## Abstract

Early and progressive cortico-striatal circuit alterations have been widely characterized in Huntington's disease (HD) patients. Cortical premotor area, M2 cortex in rodents, is the most affected cortical input to the striatum from early stages in patients and is associated to the motor learning deficits present in HD mice. Yet, M2 cortex sends additional long-range axon collaterals to diverse output brain regions beyond basal ganglia. Here, we aimed to elucidate the contribution of M2 cortex projections to HD pathophysiology in mice. Using fMRI, M2 cortex showed most prominent functional connectivity alterations with the superior colliculus (SC) in symptomatic *R6/1* HD male mice. Structural alterations were also detected by tractography, although diffusion weighted imaging measurements suggested preserved SC structure and similar electrophysiological responses were obtained in the SC on optogenetic stimulation of M2 cortical axons. Male and female HD mice showed behavioral alterations linked to SC function, including decreased defensive behavioral responses toward unexpected stimuli, such as a moving robo-beetle, and decreased locomotion on an unexpected flash of light. Additionally, GCamp6f fluorescence recordings with fiber photometry showed that M2 cortex activity was engaged by the presence of a randomly moving robo-bettle, an effect absent in HD male mice. Moreover, acute chemogenetic M2 cortex inhibition in WT mice shift behavioral responses toward an HD phenotype. Collectively, our findings highlight the involvement of M2 cortex activity in visual stimuli-induced behavioral responses, which are deeply altered in the *R6/1* HD mouse model.

**SIGNIFICANCE STATEMENT** Understanding brain circuit alterations in brain disorders is critical for developing circuit-based therapeutic interventions. The cortico-striatal circuit is the most prominently disturbed in Huntington's disease (HD); and particularly, M2 cortex has a prominent role. However, the same M2 cortical neurons send additional projections to several brain regions beyond striatum. We characterized new structural and functional circuitry alterations of M2 cortex in HD mouse models and found that M2 cortex projection to the superior colliculus (SC) was deeply impaired. Moreover, we describe differential responses to unexpected sensory stimulus in HD mouse models, which relies on SC function. Our data highlight the involvement of M2 cortex in SC-dependent sensory processing and its alterations in HD pathophysiology.

## Introduction

Huntington's disease (HD) is a neurodegenerative disorder characterized by devastating motor symptoms, including chorea, and preceded by cognitive and psychiatric disturbances. HD pathology is caused by a CAG trinucleotide expansion (>36 repeats) in the huntingtin gene (Huntington's Disease Collaborative Research Group, 1993) and results in a progressive degeneration of medium-sized spiny neurons in the caudate and putamen (striatum). The cortex is the main input to the striatum, and an early and progressive cortico-striatal disconnection has been extensively demonstrated by several studies in HD patients (Unschuld et al., 2012; Dogan et al., 2015) and animal models (Cepeda et al., 2007; Veldman and Yang, 2018). In HD patients, the most affected structural and functional striatal connections arise from cortical premotor areas, analogous to the secondary motor (M2) cortex in rodents. Notably, these affectations appear many years before the onset of motor symptoms in HD carriers (Unschuld et al., 2012; Shaffer et al., 2017; Estevez-Fraga et al., 2021; Johnson et al., 2021) and are profoundly impaired in animal models (Hintiryan et al., 2016; Creus-Muncunill et al., 2019; Fernández-García et al., 2020). However, it is unclear whether dysregulated information flow from the M2 cortex in HD affects only cortico-striatal functions or could have an additional impact in the remaining long-distance output nuclei.

Cortico-striatal circuits are required for motor learning processes (Costa et al., 2004; Cao et al., 2015; Kupferschmidt et al., 2017), and M2 cortex projection to the striatum has been associated to the motor learning deficits that characterize HD mice (Fernández-García et al., 2020). Furthermore, M2 cortex is involved in a variety of functions other than motor learning (Cao et al., 2015), such as decision-making (Sul et al., 2011) and perceptual behavior (Duan et al., 2021), as widely reviewed (Barthas and Kwan, 2017; Yang and Kwan, 2021). In this regard, pyramidal tract neurons from M2 cortex project ipsilaterally to striatum and also send collateral axons to the thalamus, subthalamic nucleus, superior colliculus (SC), pons, and spinal cord (Zhang et al., 2016; Economo et al., 2018; Gerfen et al., 2018). Yet, the involvement of M2 cortex circuitry to HD pathology has not been explored beyond basal ganglia.

In the present work, we aimed to determine the contribution of additional M2 cortex circuits to HD pathology using the *R6/1* HD mouse model. First, we mapped alterations in M2 cortex functional connectivity with the rest of the brain in WT and HD mice, by using *in vivo* resting-state fMRI techniques. Then, we selected the SC as key M2 cortex target brain region and studied the structural connectivity of M2 cortex–SC pathway by diffusion MRI analysis. To determine M2 cortex–SC functional alterations in HD mice, we took advantage of optogenetic tools, fiber photometry, chemogenetics, and SC-dependent behavioral responses to unexpected visual stimuli, such as the presence of a moving robo-beetle (Almada et al., 2018) and an unexpected flash of light (Liang et al., 2015). Together, our results show the involvement of M2 cortex activity in visual stimuli-induced behavioral responses and that the M2 cortex activity is deeply impaired in the *R6/1* HD mouse model.

## Materials and Methods

### Animals

Experiments were performed using *R6/1* transgenic mice (B6CBA background; RRID:IMSR_JAX:002809) expressing the exon 1 of *mutant huntingtin* with ∼145 CAG repeats at the time of experiments and their age-matched WT littermate as controls. Mice were acquired from The Jackson Laboratory and housed together in groups of mixed genotypes with access to food and water *ad libitum* in a colony room kept at 19°C-22°C and 40%-60% humidity, under a 12:12 h light/dark cycle. Genotypes were determined by PCR from ear biopsy, mice randomly assigned to experimental groups, and data were recorded for analysis by microchip mouse number.

Male and female mice were tested at 12 weeks of age for presymptomatic behavioral screening; male mice were tested in experiments during symptomatic phase (∼20-week-old). When required, stereotaxic surgeries were performed 4 weeks before testing (16-week-old mice). A power analysis was performed for fMRI and the behavioral experiments based on previous data from the laboratory, considering power of 0.8, α of 0.05, and SD of 20%, which leads to *N* = 12 for behavior. *N* values (biological replicates) are given throughout the manuscript in the figure legends. Each experiment was performed once. All animal procedures were conducted in accordance with the Spanish RD 53/2013 and European 2010/63/UE regulations for the care and use of laboratory animals and approved by the animal experimentation Ethics Committee of the Universitat de Barcelona and Generalitat de Catalunya.

### MRI acquisition

Experiments were blindly performed on a 7.0T BioSpec 70/30 horizontal animal scanner (Bruker BioSpin), equipped with an actively covered gradient structure (400 mT/m, 12 cm inner diameter). To evaluate connectivity between ROIs, mice (*n* = 11 WT, *n* = 13 *R6/1*) at 17-20 weeks of age underwent structural T2-weighted imaging, diffusion weighted imaging (DWI), and rs-fMRI. Animals were placed in a Plexiglas holder in a supine position and were fixed using tooth and ear bars and adhesive tape; a combination of anesthetic gases [medetomidine (bolus of 0.3 mg/kg, 0.6 mg/kg/h infusion) and isoflurane (0.5%)] was administered using a Plexiglas holder with a nose cone.

Proper position of the head at the isocenter of the magnet was ensured with a 3D-localizer scan. T2-weighted image was obtained using a RARE sequence [effective TE = 33 ms, TR = 2.3 s, RARE factor = 8, voxel size = 0.08 × 0.08 mm^2^, slice thickness = 0.5 mm]. DWI was acquired using an EPI sequence [TR = 6s, TE = 27.7 ms, voxel size 0.21 × 0.21 mm^2^, slice thickness = 0.5 mm, 30 gradient directions with b = 1000 s/mm^2^ and 5 baseline images without diffusion weighting]. rs-fMRI acquisition was performed using an EPI sequence [TR = 2 s, TE = 19.4 ms, voxel size 0.21 × 0.21 mm^2^, slice thickness = 0.5 mm]; 420 volumes were acquired and the total scan time was 14 min. After completion of the imaging session, atipamezol (Antisedan, Pfizer) and saline were injected to reverse the sedative effect and compensate fluid loss.

### Functional connectivity analysis

Seed-based analysis was performed as described previously (Fernández-García et al., 2020) to evaluate functional connectivity of the M2 cortex with the rest of the brain. Preprocessing of the rs-fMRI data included: slice timing, spatial realignment using SPM8 for motion correction, elastic registration to the T2-weighted volume using ANTs for EPI distortion correction (Avants et al., 2008), detrend, smoothing (FWHM = 0.6 mm), frequency filtering of time series between 0.01 and 0.1 Hz, and regression by motion parameters using NiTime (http://nipy.org/nitime).

Brain parcellation was defined according to the MRI-based atlas of the mouse brain (Ma et al., 2008). The sensorimotor cortex defined in the original atlas was manually divided into somatosensory cortex (Som), orbitofrontal cortex (OFC), primary motor (M1) cortex, M2 cortex, cingulate cortex (Cg), mPFC, and retrosplenial cortex (RS). The atlas template was elastically registered to each subject T2-weighted volume using ANTs (Avants et al., 2008) and finally applied to the label map to obtain specific parcellation for each animal.

Brain parcellation was registered from T2-weighted to resting-state fMRI images. The left M2 cortex was selected as seed region for seed-based analysis. Extracted average time series in the M2 cortex was correlated with the time series of all the voxels within the brain, resulting in a connectivity map containing the value of the correlation between the BOLD signal time series in each voxel with the seed time series. Seed to region connectivity was calculated as the mean value of the correlation map in each region, considering only positive correlations.

### Structural connectivity analysis

The fiber tracts connecting M2 cortex with SC were identified and characterized based on automatically identified regions in the T2-weighted image and tractography results from DWI. Diffusion tensor images (DTI) were estimated, and standard DTI metrics were computed, including fractional anisotropy (FA), mean diffusivity (MD), axial diffusivity (AD), and radial diffusivity (RD). To estimate the fiber tracts, DWI was preprocessed, including eddy-current correction using FSL (Jenkinson et al., 2012), denoising (Coupe et al., 2008), and bias correction (Tustison et al., 2010). The five baseline images were averaged and registered to the T2-weighted image to correct for EPI distortions. Whole-brain tractography was performed using a deterministic algorithm based on constrained spherical deconvolution model, considering as seed points the voxels where FA > 0.1. The same threshold was defined as stop criterion for the algorithm. Once the fiber tracts were identified, its average FA, MD, RD, and AD were computed.

### Stereotaxic surgery

We used different adeno-associated virus (AAV): AAV-ChR2 virus construct under CamKII promotor (AAV1-CaMKIIa-hChR2(H134H)-eYFP-WPRE.hGH; catalog #AV-1-26969P, University of Pennsylvania-Penn Vector core) was used for optogenetic modulation; AAV-GCaMP6f virus construct under SYN promotor (AAV9-Syn-GCaMP6f-WPRE-SV40; Addgene catalog #100837-AAV9) was used for fiber photometry analysis. AAV-hM3D(Gi) virus under hSyn promotor (AAV5-hSyn-hM3D(Gi)-mCherry; catalog #50475-AAV5) was used for chemogenetic inhibition. Virus production, amplification, and purification were performed by University of Pennsylvania-Penn Vector Core and Addgene (titers: ∼1 × 10^12^ genomic particles/ml).

Stereotaxic surgery was performed under isoflurane anesthesia (5% induction and 1.5% maintenance) in 16-week-old mice. Meloxicam (2 mg/kg s.c.) was injected before the surgery to avoid pain and reduce inflammation. A volume of 0.5 µl of corresponding viral constructs was injected in M2 cortex (mm from bregma and dura matter: 2.46 AP, ±1 ML, −0.8 DV) using 5 µl Hamilton syringe with a 33 gauge needle at 0.1 µl/min. To allow diffusion of virus particles and avoid refluxes, the needle was left for an additional 5 min period. Animals were housed for viral expression and recovery from surgery at least 4 weeks before behavioral experiments were initiated.

For fiber photometry analysis *in vivo*, fiber-optic cannulas (MFC_400/430-0.66_1.0 mm_MF1.25_FLT; Doric Lenses) were implanted unilaterally in the left hemisphere of the M2 cortex (mm from bregma and dura matter: 2.46 AP, ±1 ML, −0.8 DV). All fiber-optic cannulas were implanted during surgery and fixed in place using dental cement.

### Behavioral assessment

All behavioral tests were performed by an experimented observer during the light phase. Animals were habituated to the experimental room for at least 1 h before testing. All testing apparatus were cleaned with water and dried between tests and animals.

#### Beetle Mania Task (BMT)

The BMT assesses both passive and, in particular, active fear responses of the mice to an unexpected moving beetle (Nano Nitro, Hexbug) (Heinz et al., 2017; Almada et al., 2018). The test was performed using a white rectangular arena (15 × 40 × 30 cm) with dim light (∼20 lux). Briefly, the test comprises two successive phases of 5 min: during the habituation phase, mice freely explored the arena. During this time, distance traveled and rearing times were scored using the SMART 3.0 software (Panlab). In the testing phase, confrontations with the erratically moving robo-beetle were scored. The robo-beetle was introduced to the arena at maximal distance to the mouse and the following behavioral responses analyzed: (1) escape, mice quickly jump (flight) with accelerated speed in the direction opposite to the beetle's movement vector; (2) tolerance, ignorance of the robo-beetle after physical contact; (3) approach, mice follow the robo-beetle in close vicinity; (4) avoidance, mouse walks in opposite direction to the robo-beetle; and (5) total responses, the sum of all the events evaluated. Escape events were normalized to total responses, whereas tolerance, approach, and avoidance were normalized to the sum of these three parameters, without the escape events, as previously described (Almada et al., 2018).

#### Light-induced locomotion

The task assesses light-triggered behaviors in freely moving mice and was performed based on Liang et al. (2015). The arena consisted of a narrow and long hall (8 × 150 × 30 cm^3^) dimly illuminated (∼20 lux) with a bulb with bright and white light placed at equity distance from the beginning and the end of the corridor. Mice were placed in one site of the hall and freely walked to the opposite site; then, when passed below the bulb, it triggered an ∼1 s light flash above the animal. Each animal was tested for 5 trials, with intertrial intervals of ∼20 min, and scored: Time 1, time mice walked from the beginning of the hall to the bulb; and Time 2, time mice walked from the bulb to the end of the corridor.

### Fiber photometry

Changes of neuronal activity were assessed using the GCamp6f fluorescent calcium indicator and normalized using isosbestic fluorescent recordings (405 nm), using a custom-made fiber photometry system obtained from Doric Lenses. We used the free Doric Neuroscience studio Software to control the Doric console and LED drivers. A 465 nm LED (CLED_465, Doric Lenses) modulated at 241.58 MHz and a 405_nm LED (CLED_405, Doric Lenses) modulated at 572.21 MHz were directed and coupled into a personalized fluorescence mini cube (iFMC7, Doric Lenses) through 1 m attenuator fiber-optic patchcords (400 µm core, 0.48 NA). Combined 465_nm and 405_nm light was launched to a pigtailed (400 µm core, 0.57 NA) fiber-optic rotary joint (Doric Lenses) connected to a low autofluorescence mono fiber-optic patchcord (400 µm core, 0.57 NA) allowing freely moving tests with mice, and finally mated to the implanted fiber-optic cannula (see stereotaxic surgery) via a black covered Zicronia sleeve. Emitted fluorescence travels back through the mini cube and spectra, detected and amplified by the Fluorescence Detector Amplifier from Doric. Data were sent to the fiber photometry console. Fiberoptic patchcords were bleached for 3 h before each experiment. Mice were connected to the optical fiber 5 min before the task and the 465 and 405 LED were turned on to ensure stable photometric recordings.

Raw and demodulated 465 nm and 405 nm fluorescent intensity data were recorded at 12,000 Hz in freely moving mice using the free Doric Neuroscience studio Software. Demodulated data were further processed by a custom-made software provided by Metofico and based on MATLAB. Briefly, data were downsampled to 1 Hz and artifacts removed using a custom digital filter. Fluorescent increments were normalized to baseline (ΔF/F_0_), consisting in the average fluorescence in 1 min window before the introduction of the beetle. To correct movement artifacts, normalized 405 nm signal was subtracted from the normalized 465 nm.

### Chemogenetic inhibition

Inactivation of M2 cortex was done using the system of designer receptor exclusively activated by designer drug (DREADD). According to Wang et al. (2020), mice expressing AAV5-hSyn-hM3D(Gi)-mCherry in M2 cortex were intraperitoneally injected ∼40 min before the BMT with clozapine-N-oxide (CNO, BML-NS105-0025 Enzo) (1.5 mg/kg) or saline as control.

### Optogenetic stimulation and multielectrode array (MEA) recordings

Electrophysiological recordings were performed as previously described (Fernández-García et al., 2020; Kim et al., 2020). Briefly, coronal sections of mouse brain were obtained on a vibratome at 350 µm thickness in ice-cold aCSF oxygenated (95% O_2_, 5% CO_2_) and then transferred for 15 min to an oxygenated 32°C recovery solution. Slices were then transferred to oxygenated aCSF at room temperature for at least 1 h before recording.

Electrophysiological data were recorded by a blind experimenter using a MEA set-up from Multi Channel Systems MCS. MC Stimulus and MC Rack software from Multi Channel Systems were used for stimulation, recording, and signal processing. We assessed the correct position of the slices on the electrode field using a digital camera during the recording.

Field postsynaptic currents (fPSCs) were recorded in the lateral SC in response to the optogenetic stimulation of M2 cortical afferents with 1 ms 473 nm light pulses of increasing intensities (0.024, 0.033, 0.911, 4.23, 10.2, 19.8, 44.8, and 79.0 mW/mm^2^), delivered by a 473 nm diode pumped solid state laser (Laserglow). The laser was approximately placed in the surface of the slide at ∼−4.04 posterior to bregma, −1.75 ventral from the skull, and ±1.45 lateral from the middle, corresponding to lateral SC. Evoked fPSCs responses in the SC were analyzed after trains of 1 ms light stimuli.

### Immunohistochemistry

Mice were killed by cervical dislocation and brains were postfixed with 4% PFA for immunohistochemical analysis, dehydrated in a PBS/sucrose gradient [from 15% (48 h postmortem) to 30% (32 h postmortem)] with 0.02% sodium azide, snap frozen and stored at −20°C. Sections (30 µm) were cut on a vibratome (Leica VT1000S) and preserved in PBS with 0.02% sodium azide at 4°C. Anti-GFP (1:500, Invitrogen, #11122, RRID:AB_221569) antibody was used to evaluate expression of AAV-YFP, AAV-GCAMP6f. Free-floating sections were washed in PBS and permeabilized and blocked for 15 min in PBS containing 0.3% Triton X-100 and 3% normal goat serum (Pierce Biotechnology). Sections were washed again in PBS and incubated overnight at 4°C with primary antibodies. Brain slices were washed, incubated for 2 h with the secondary antibody (1:200 goat anti-rabbit Cy3, Jackson ImmunoResearch Laboratories, catalog #111-165-003, RRID:AB_2338000, and mounted on microscope slides using DAPI Fluoromount-G, Southern Biotechnology). Fluorescence images were acquired by an epifluorescence microscope (DMI6000 Leica). No signal was detected in absence of primary antibody. Mice with no GFP expression observed, indicating no expression of AAVs, were discarded.

### Statistical analysis

All the results were expressed as mean ± SEM. Data from individual mouse was represented by single points when possible. GraphPad Prism version 8.0.0. Software was used for statistical analysis. Student's *t* test and the one-way and the two-way ANOVA analysis, followed by Bonferroni *post hoc* test were performed when appropriate, and indicated in Results and/or figure legends. Values of *p* < 0.05 were considered as statistically significant.

## Results

### The M2 cortex is functionally disconnected with the SC in HD mice

**Figure 1.**
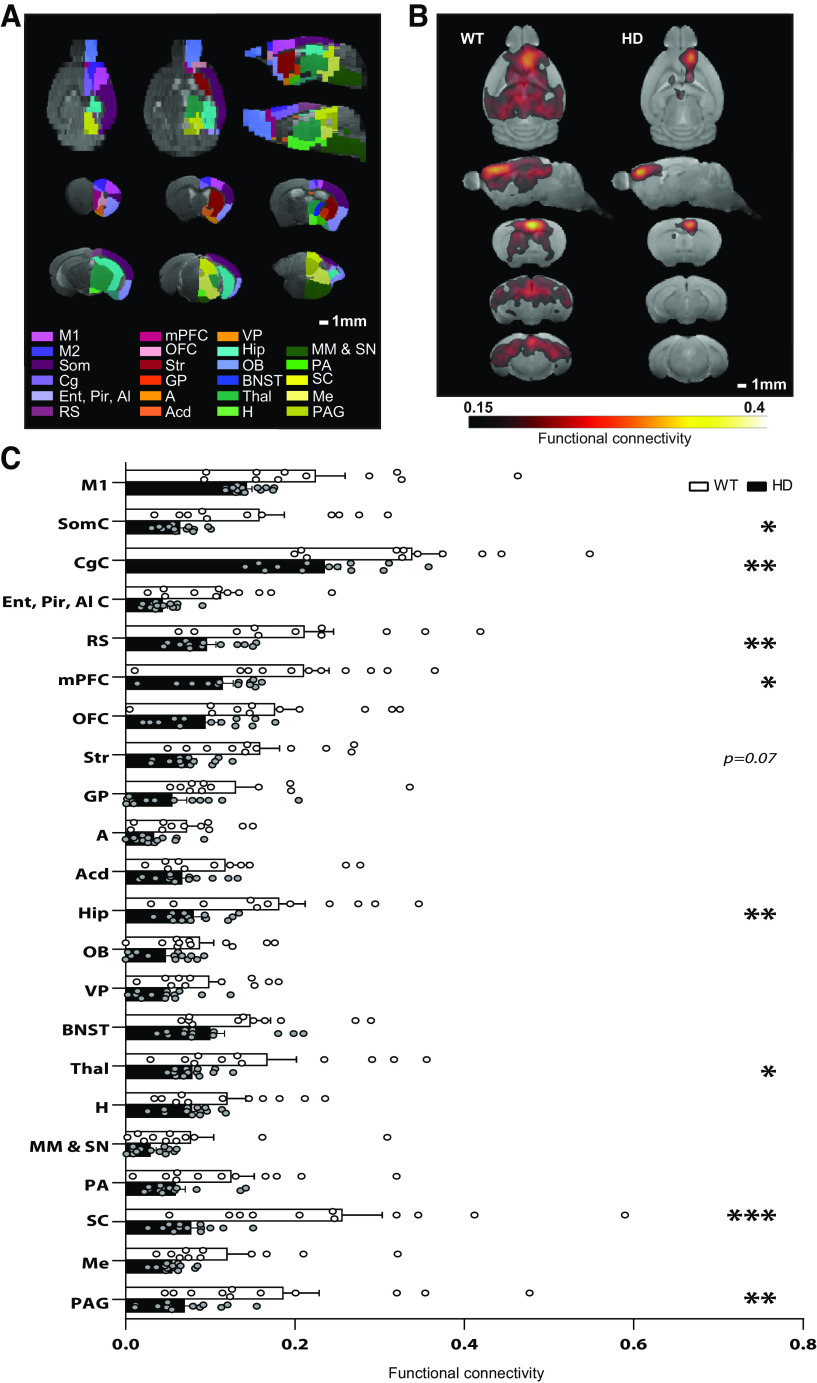
Functional connectivity analysis using rs-fMRI indicates the SC as the brain region most severely disconnected from M2 cortex in 20-week-old HD mice. ***A***, Anatomical regions for functional connectivity analysis based on atlas automatic parcellation. ***B***, Average functional connectivity maps of left M2 cortex in WT and the *R6/1* mouse model of HD. Color maps represent the average correlation value when >0.15. ***C***, Average functional connectivity of the left M2 cortex with all brain areas analyzed from the left hemisphere are represented. Average of the seed-based correlation map in each area represents functional connectivity between the M2 cortex and each region. Each point represents data from an individual mouse. Two-way ANOVA with genotype and brain region as factors was performed, followed by Bonferroni *post hoc* comparisons test. Data are mean ± SEM (WT *n* = 11 and HD *n* = 13 mice; 17-week-old mice). *p < 0.05, **p < 0.01, ***p < 0.001, HD versus WT. A, Amygdala; Acd, nucleus accumbens; BNST, bed nucleus of the stria terminalis; Cg, cingulate cortex; Ent, entorhinal cortex; Pir, piriform cortex; AI, agranular insular cortex; GP, globus pallidus; Hip, hippocampus; H, hypothalamus; MM, medial mammillary nucleus; SN, substantia nigra; Me, mesencephalic nucleus; mPFC, medial prefrontal cortex; OB, olfactory bulb; OFC, orbitofrontal cortex; PAG, periaqueductal gray; PA, preoptic area; M1, primary motor cortex; M2, secondary motor cortex; RS, retrosplenial cortex; Som, secondary somatosensory cortex; Str, striatum; Thal, thalamus; VP, ventral pallidum.

To investigate M2 cortex connectivity alterations in HD, we used rs-fMRI and analyzed functional connectivity using seed-based analysis between the M2 cortex and 22 automatically identified brain regions covering the left brain hemisphere in ∼20-week-old WT and *R6/1* mice (Fig. 1). M2 cortex showed the highest functional connectivity with Cg and SC in WT mice. In addition, functional connectivity between the M2 cortex and all the brain regions analyzed was reduced in HD mice compared with WT. Two-way ANOVA reported a significant genotype effect (*F*_(1,22)_ = 11.9; *p* = 0.002), brain region effect (*F*_(21 462)_ = 35.9, *p* < 0.0001), and region × genotype interaction effect (*F*_(21,462)_ = 3.6, *p* < 0.0001). The left M2 cortex of HD mice, compared with WT, showed significantly reduced functional connectivity with left SC (*p* < 0.0001), but also with left periaqueductal gray (PAG; *p* = 0.001), thalamus (Thal; *p* = 0.05), hippocampus (Hip; *p* = 0.01), RS (*p* = 0.001), Cg (*p* = 0.007), Som (*p* = 0.02), and mPFC (*p* = 0.02), as shown by Bonferroni *post hoc* test (Fig. 1). Thus, our results highlight that M2 cortex functional connectivity deficits in symptomatic HD mice involves several cortical, hippocampal, and non-basal ganglia brain regions, with most prominent changes with the SC.

### Structural alterations in M2 cortex projection to SC in HD mice

**Figure 2.**
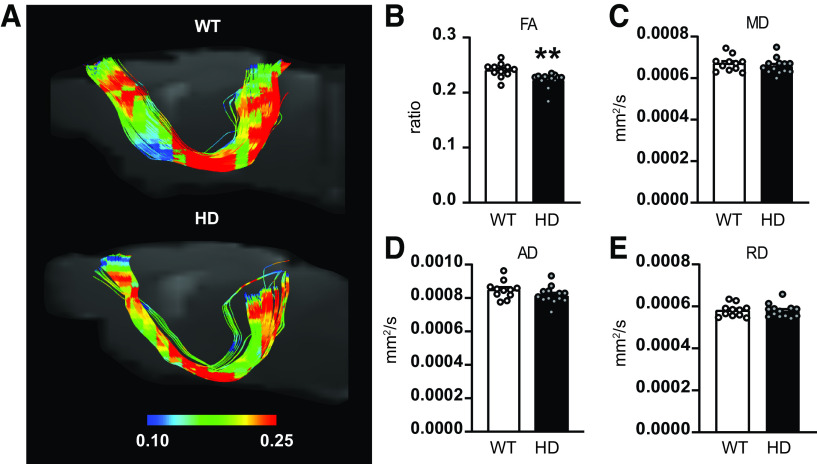
Diffusion metrics indicate structural alterations in M2 cortex–SC tracts of HD mice. ***A***, Representative tracts connecting the M2 cortex area with the SC in WT and the *R6/1* mouse model of HD. M2 cortex and SC areas were automatically delimited according to atlas. ***B***, ***E***, Diffusion metrics analysis of tracts between M2 cortex and SC includes (***B***) FA, (***C***) MD, (***D***) AD, and (***E***) RD. Each point represents data from an individual mouse. Unpaired Student's *t* test was performed. Data are mean ± SEM (WT *n* = 11 and HD *n* = 13 mice; ∼20-week-old mice). **p < 0.01.

**Figure 3.**
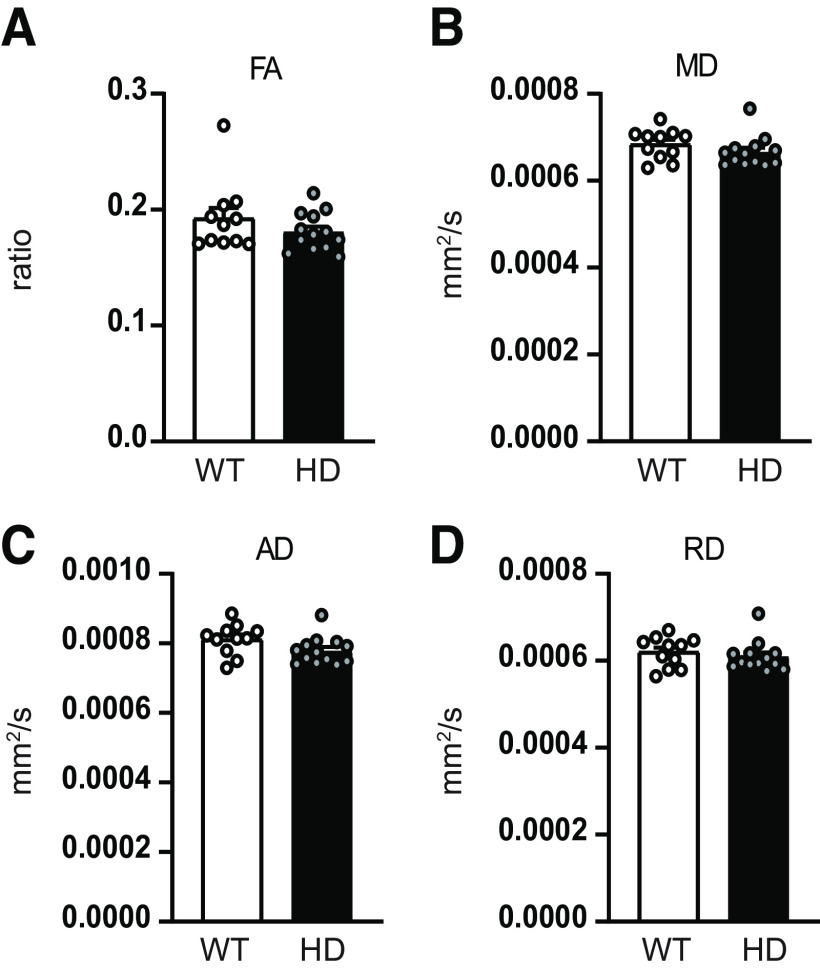
Regional diffusion metrics indicate preserved microstructure in the SC of HD mice. ***A***, FA, (***B***) MD, (***C***) AD, and (***D***) RD were measured in the SC of 20-week-old WT and *R6/1* mice. Each point represents data from an individual mouse. Unpaired Student's *t* test was performed. Data are mean ± SEM (WT *n* = 11 and HD *n* = 13 mice; ∼20-week-old mice).

To evaluate whether functional connectivity deficits observed in M2 cortex–SC circuit were associated to structural alterations, we estimated the M2 cortex–SC tracts from DWI and measured FA as well as MD, AD, and RD diffusivity (Fig. 2). Our data indicate that structural alterations are also present in the M2 cortex–SC tract. We reported significant decreased FA values (Fig. 2*B*) when comparing HD with WT mice, while MD, AD, and RD remained similar between genotypes (Fig. 2*C–E*). In addition, we analyzed DTI metrics within the SC to exclude possible regional microstructural alterations (Fig. 3). All the evaluated diffusion metrics measured in the SC were similar between WT and HD mice, suggesting that microstructure alterations in the M2 cortex–SC are related to the projection between both brain regions, but not to the SC alone.

### Optogenetic stimulation of M2 cortical axons in the SC evoked similar responses in WT and HD mice

**Figure 4.**
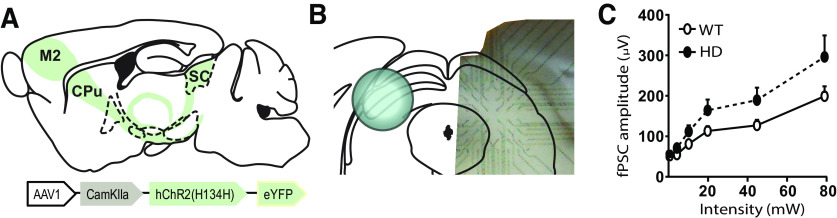
Optogenetic stimulation of M2 cortex terminals in the SC evoked similar electrophysiological responses in WT and HD mice. ***A***, Schematic representation of AAV-CamKII-ChR2-YFP construct injection at M2 cortex. ***B***, Representative MEA location in coronal slices containing the SC with axons from M2 cortex expressing ChR2. ***C***, Amplitude of fPSC triggered by increasing light intensities in the SC of WT and HD mice. Data are mean ± SEM (WT *n* = 7 and HD *n* = 8 mice; 21-week-old mice).

To elucidate whether SC respond to M2 cortex activity in both WT and HD mice, we performed MEA recordings in the deep layers of the lateral part of the of the SC (dlSC) in WT and HD mice, which were previously injected in the M2 cortex with the AAV-CamKII-ChR2-YFP construct (Fig. 4). A normalized input-output assay was created by recording fPSC in the dlSC induced by increasing light intensities. Our results revealed that optical stimulation of M2 cortical axons in the dlSC evoked fPSC in both WT and HD mice. Two-way ANOVA showed a significant light intensify effect (*F*_(7,84)_ = 34.5, *p* < 0.0001), although neither genotype effect (*F*_(1,12)_ = 3.0, *p* = 0.1) nor genotype × light intensity interaction (*F*_(7,84)_ = 1.9, *p* = 0.08) were found.

### Impaired SC-associated behavioral functions in symptomatic HD mice

The SC receives and integrates visual information to control reflex movements, such as stimulus orienting (approach response) or defensive movements (avoidance or flight) (Wurtz and Albano, 1980; Sparks, 1986; Dean et al., 1989). To examine whether SC-dependent behaviors were affected in HD mice, we selected two tasks known to involve SC function in rodents: BMT (Almada et al., 2018) and the visually induced locomotor behavior (Liang et al., 2015).

**Figure 5.**
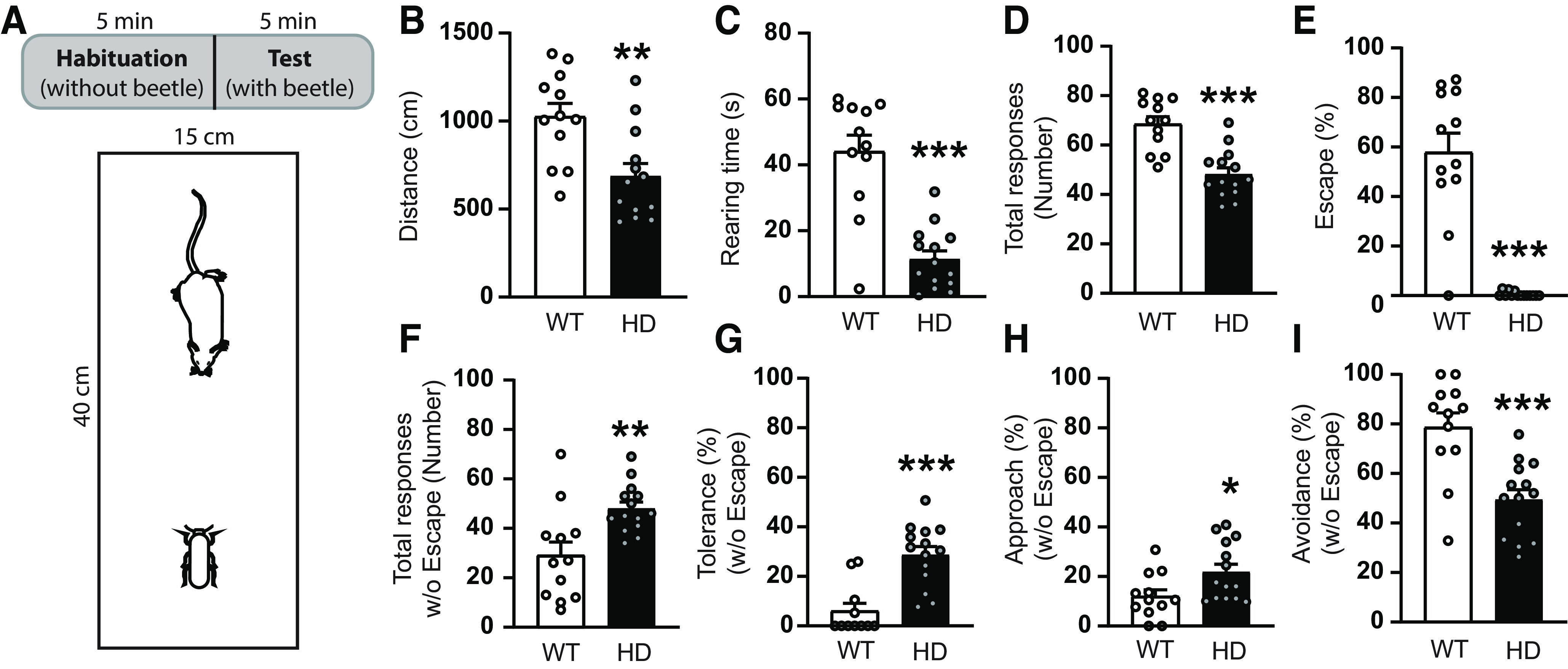
Behavioral responses toward a randomly moving robo-beetle differ between WT and HD mice at symptomatic stages. ***A***, Schematic representation of the BMT protocol, including 5 min habituation and 5 min test phases in a rectangular arena. ***B***, ***C***, During the habituation phase, (***B***) distance traveled and (***C***) rearing time were measured. ***D-I***, During the test phase, (***D***) total induced responses to the robo-beetle are represented. Percentages of (***E***) escape responses, (***F***) total responses excluding escape, and percentage of (***G***) tolerance, (***H***) approach, and (***I***) avoidance responses when excluding the escape response were analyzed. Each point represents data from an individual mouse. Unpaired Student's *t* test was performed. Data are mean ± SEM (WT *n* = 12 and HD *n* = 14 mice; 21-week-old mice). *p < 0.05. **p < 0.01. ***p < 0.001.

In the BMT (Fig. 5), the behavioral responses to a randomly moving robo-beetle are evaluated. During the habituation phase of the test, mice were allowed to explore the arena for 5 min and locomotion and spontaneous exploration, such as rearing time, was significantly decreased in HD mice compared with WT (Fig. 5*B*,*C*), as previously described (Pépin et al., 2016). During the testing phase, when mice were confronted with the robo-beetle, HD mice showed a significant decrease in total number of responses to the robo-beetle (Fig. 5*D*). Then, we evaluated the type of response toward the robo-beetle contact, and we observed that the major response in WT mice was to escape from the robo-beetle, compared with the other evaluated responses, such as tolerance, approach, or avoidance. Interestingly, HD mice show little or non-escape response, as demonstrated by the strongly reduced total percentage of escape response (Fig. 5*E*). We further evaluated the percentage of responses when excluding escape behavior, as previously described (Almada et al., 2018) and found that number of total responses from HD mice toward the robo-beetle was significantly higher compared with WT when obviating escape responses (Fig. 5*F*). Of those, HD mice had significantly increased both tolerance (Fig. 5*G*) and approach responses (Fig. 5*H*). Finally, although avoidance behavior is the preferred response to the robo-beetle in both genotypes (when excluding escape responses), HD mice showed significantly reduced avoidance responses compared with WT mice (Fig. 5*I*). Together, our data highlight that the choice of response to unexpected threatening beetle differs in HD compared WT mice, suggesting alterations in coupling of visual inputs and thread responding.

**Figure 6.**
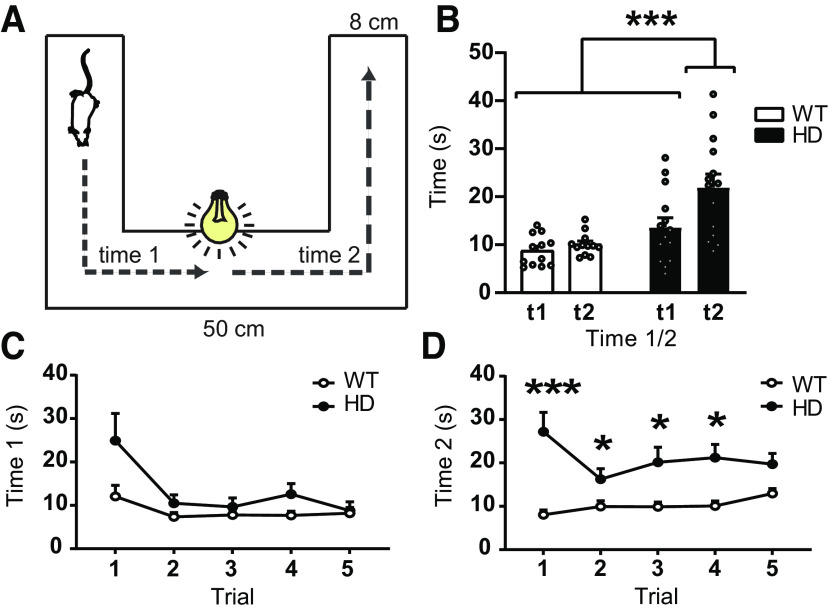
The behavioral response to unexpected flash of light is altered in symptomatic HD mice. ***A***, Schematic representation of the task protocol. A mouse will travel through a corridor, and a sudden flash of light is opened when the mouse reaches the center. ***B***, Plot of average time to travel until the center (Time 1, t1) and from the center to the end of the corridor (Time 2, t2). Each point represents the average of the 5 trials from an individual mouse. ***C***, ***D***, Graphs showing the measures of Time 1 (***C***) and Time 2 (***D***) over the 5 trials. Two-way ANOVA with genotype and time or trials as factors was performed and followed by Bonferroni *post hoc* test comparisons. Data are mean ± SEM (WT *n* = 12 and HD *n* = 14 mice; 21-week-old mice). *p < 0.05. ***p < 0.001.

Next, we assessed light-triggered locomotor behaviors in response to unexpected stimuli from the upper-visual field (Liang et al., 2015). We compared the response to a sudden flash of white light (∼1 s duration) in the center of a dark corridor between genotypes (Fig. 6). We observed that a sudden light induced a short freezing (arrest behavior) in WT mice (data not shown). Although we did not observe reduction in time to travel through the corridor after the light in WT mice as previously described (Liang et al., 2015), we found this time increased in HD mice. Two-way ANOVA reported a significant genotype effect (*F*_(1,24)_ = 11.0, *p* = 0.003), Time 1/Time 2 effect (*F*_(1,24)_ = 20.8, *p* = 0.0001), and interaction effect (*F*_(1,24)_ = 10.9, *p* = 0.003). Bonferroni *post hoc* analysis showed that locomotion was not affected in WT mice by the sudden light (Fig. 6*B*), whereas it was significantly increased in HD mice and consistent among trials (Fig. 6*C*,*D*). On the one hand, time to reach the center of the corridor was affected by the trial number, as shown by two-way ANOVA with a significant trial effect (*F*_(4,85)_ = 6.8, *p* < 0.0001), but neither genotype effect (*F*_(1,24)_ = 4.3, *p* = 0.05) nor interaction (*F*_(4,85)_ = 6.8, *p* = 0.09) (Fig. 6*C*). On the other hand, time from the center of the corridor to the end was increased in all trials in HD mice, as shown by two-way ANOVA significant genotype effect (*F*_(1,24)_ = 15.8, *p* = 0.0006) and interaction (*F*_(4,85)_ = 3.8, *p* = 0.007), but no trial effect (*F*_(4,85)_ = 1.1, *p* = 0.3) (Fig. 6*D*). Therefore, our data further confirm that WT and HD mice generate divergent responses in response to unexpected visual stimuli.

**Figure 7.**
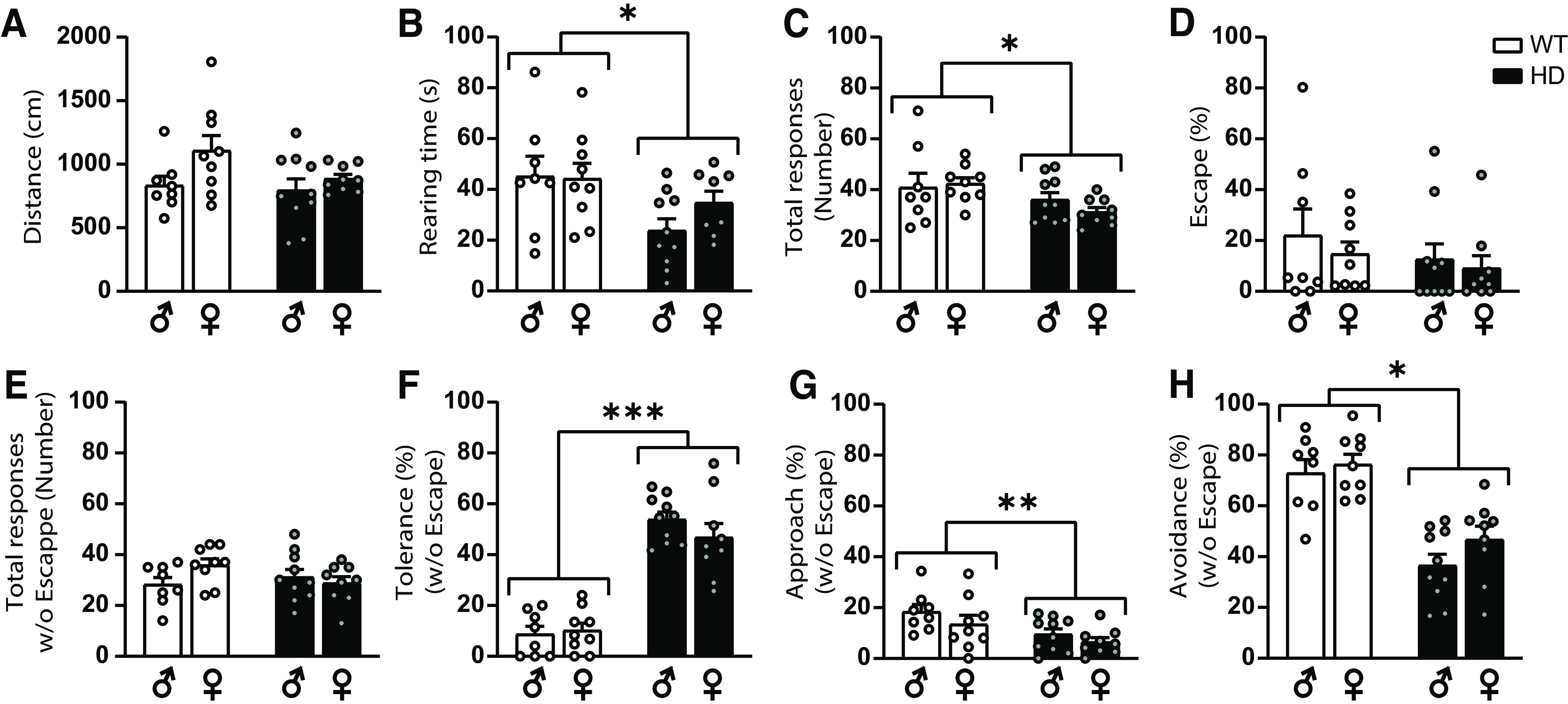
Behavioral responses toward a randomly moving robo-beetle are altered in both male and female HD mice compared with WT at 12 weeks of age. ***A***, ***B***, During the habituation phase, (***A***) distance traveled and (***B***) rearing time were measured. ***C***-***H***, During the test phase, (***C***) total induced responses to the robo-beetle are represented. Percentages of (***D***) escape responses, (***E***) total responses excluding escape, and percentage of (***F***) tolerance, (***G***) approach, and (***H***) avoidance responses when excluding the escape response were analyzed. Each point represents data from an individual mouse. Two-way ANOVA with genotype and sex as factors was performed. Data are mean ± SEM (male WT *n* = 8; male HD *n* = 10; female WT *n* = 9; female HD *n* = 9 mice; 12-week-old mice). *p < 0.05. **p < 0.01. ***p < 0.001.

To exclude that gender or motor alterations could confound the present results, the BMT was performed in presymptomatic male and female mice at 12 weeks of age, when no motor coordination symptoms are yet manifested (Canals et al., 2004; Anglada-Huguet et al., 2014) (Fig. 7). Our results confirm that at this stage locomotor activity is not altered, while spontaneous behavior such as rearing is already reduced in HD mice compared with control. Moreover, altered responses toward the randomly moving robo-beetle were similarly present at this stage in both sexes, with reduced number of responses, similar total responses without escape, increased tolerance and reduced avoidance and approach responses in HD mice compared with control WT mice (Fig. 7). In sum, SC-related behaviors are altered in HD mice from presymptomatic stages.

### Visual stimuli engage neuronal activity in the M2 cortex of WT but not HD mice

**Figure 8.**
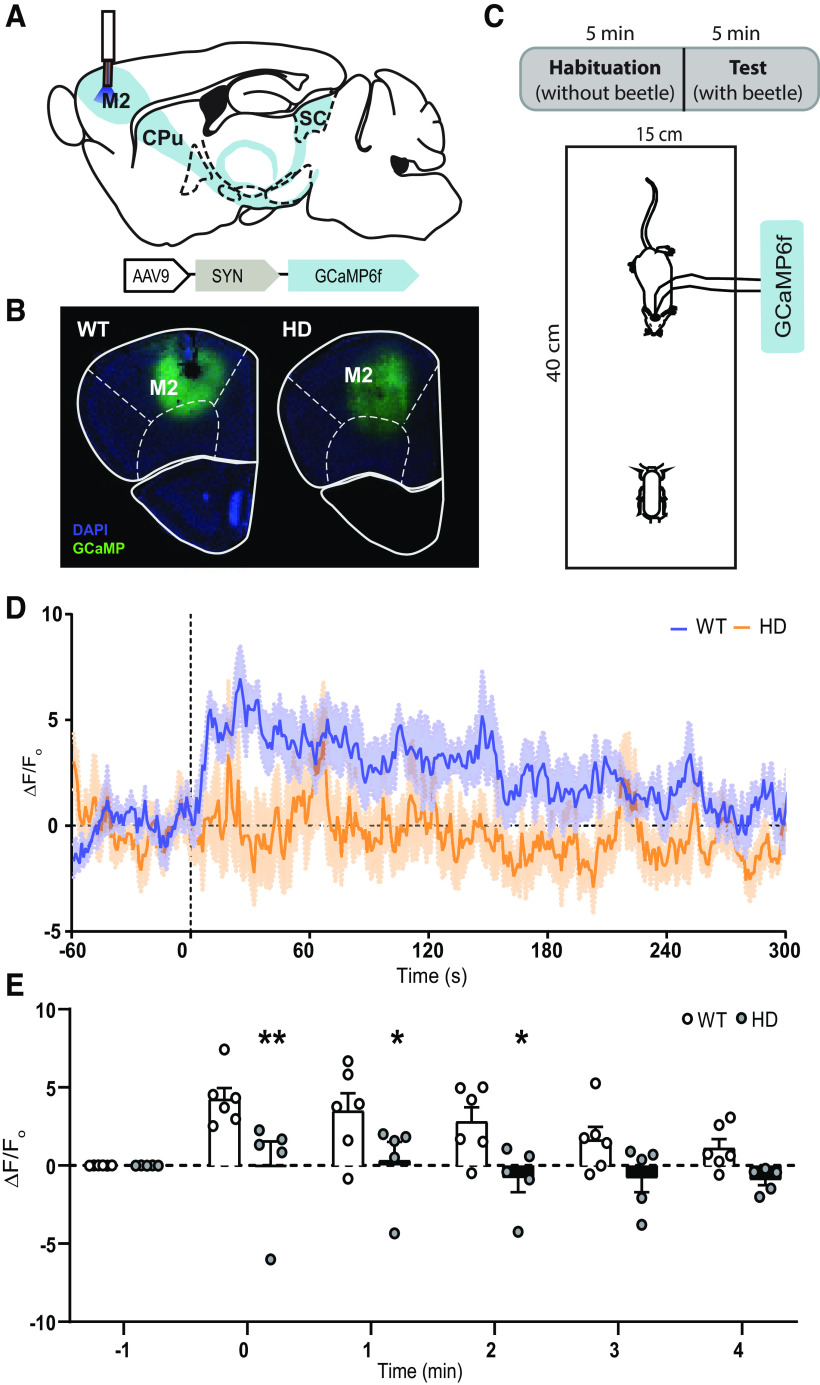
The presence of visual stimuli activates M2 cortex neurons in WT but not HD mice, shown by fluorescent calcium recordings using fiber photometry *in vivo*. ***A***, Schematic representation of fiber-optic cannula implant and representative image of the injection of the AAV-GCaMP6f construct at M2 cortex. ***B***, Representative fluorescent image showing DAPI (blue), GCaMP6f expression (green), and fiber-optic cannula implant in the M2 cortex of WT (left) and HD (right) mice. ***C***, BMT protocol, including 5 min habituation and 5 min test. ***D***, Mean photometric recordings of M2 cortex fluorescent calcium signal 1 min before and 5 min after the introduction of a moving robo-beetle to the arena in WT and symptomatic HD mice. Increases in fluorescent signal are normalized by the respective baseline for each mouse. ***E***, Average of normalized fluorescence signal is computed and represented for each minute. Each point represents data from an individual mouse. Two-way ANOVA analysis with genotype and time as factors was performed and followed by Bonferroni *post hoc* test comparisons. Data are mean ± SEM (WT *n* = 6 and HD *n* = 5 mice; 20-week-old mice). *p < 0.05, **p < 0.01, versus WT.

Because neurons from the M2 cortex project to the dlSC and are associated to lower visual stimulus (Comoli et al., 2012), we hypothesize that the activity of M2 cortex neurons might contribute to alterations in the BMT observed in HD mice. To prove this, we assessed whether M2 cortex activity correlated with the presence of an unexpected lower-visual stimuli, such as the presence of a robo-beetle (Fig. 8). We expressed a GCaMP6f calcium sensor in M2 cortical neurons by injecting an AAV-GCaMP6f construct (Fig. 8*A*,*B*) and recorded fluorescence during the 10 min of the BMT (Fig. 8*C*). Fluorescence signal was stable and similar between genotypes during the habituation phase, and markedly increased straight after introducing the robo-beetle in all WT mice, but not in HD mice (Fig. 8*D*).

To better quantify these results, we averaged fluorescent data, to obtain 1 min windows, and evaluated genotypes differences over time. Two-way ANOVA analysis reported a significant time effect (*F*_(5,45)_ = 4.7, *p* = 0.002), genotype effect (*F*_(1,9)_ = 7.4, *p* = 0.02), and time × genotype interaction (*F*_(5,19)_ = 3.1, *p* = 0.02) (Fig. 8*E*). Bonferroni *post hoc* analysis showed significant differences between groups during the first 3 min of the testing phase. Thus, our data confirm that M2 cortex becomes active in WT mice in the presence of an unexpected lower-visual stimuli. However, this neuronal engagement is lacking in HD mice.

**Figure 9.**
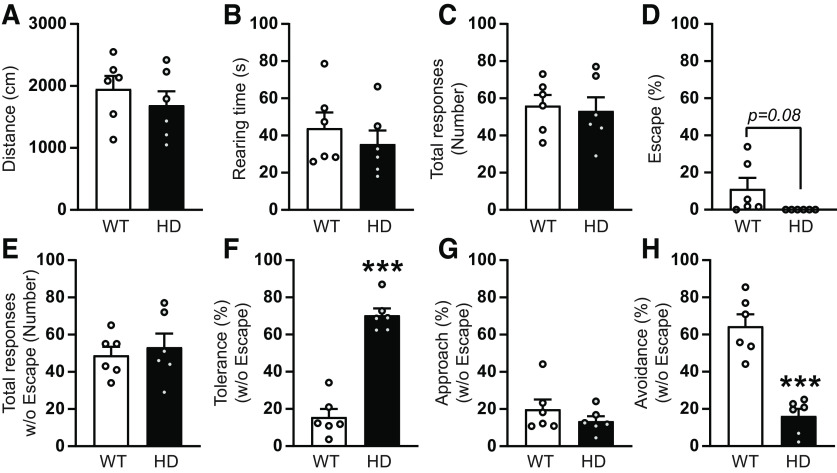
Behavioral responses during the BMT coupled to *in vivo* fiber photometry in WT and HD mice, related to Figure 8. ***A***, ***B***, During the habituation phase, (***A***) distance traveled and (***B***) rearing time were measured. ***C***-***H***, During the test phase, (***C***) total responses to the robo-bettle, (***D***) percentage of escape responses, (***E***) total responses excluding escape, and percentage of (***F***) tolerance, (***G***) approach, and **(*H***) avoidance responses when excluding the escape response were analyzed. Each point represents data from an individual mouse. Unpaired Student's *t* test was performed. Data are mean ± SEM (WT *n* = 6 and HD *n* = 6 mice; 20-week-old mice). ***p < 0.001.

Interestingly, while all WT mice engaged M2 activity, not all of them showed escape responses from the robo-beetle in this experiment (Fig. 9), suggesting that M2 cortical engagement does not exclusively reflect this specific behavioral response. Additionally, and despite the low number of mice, tolerance responses were significantly increased and avoidance responses were significantly reduced, in line with results from Figure 5.

### Acute inhibition of M2 cortical neurons in WT mice slightly shifts WT responses in the BMT toward an HD phenotype

**Figure 10.**
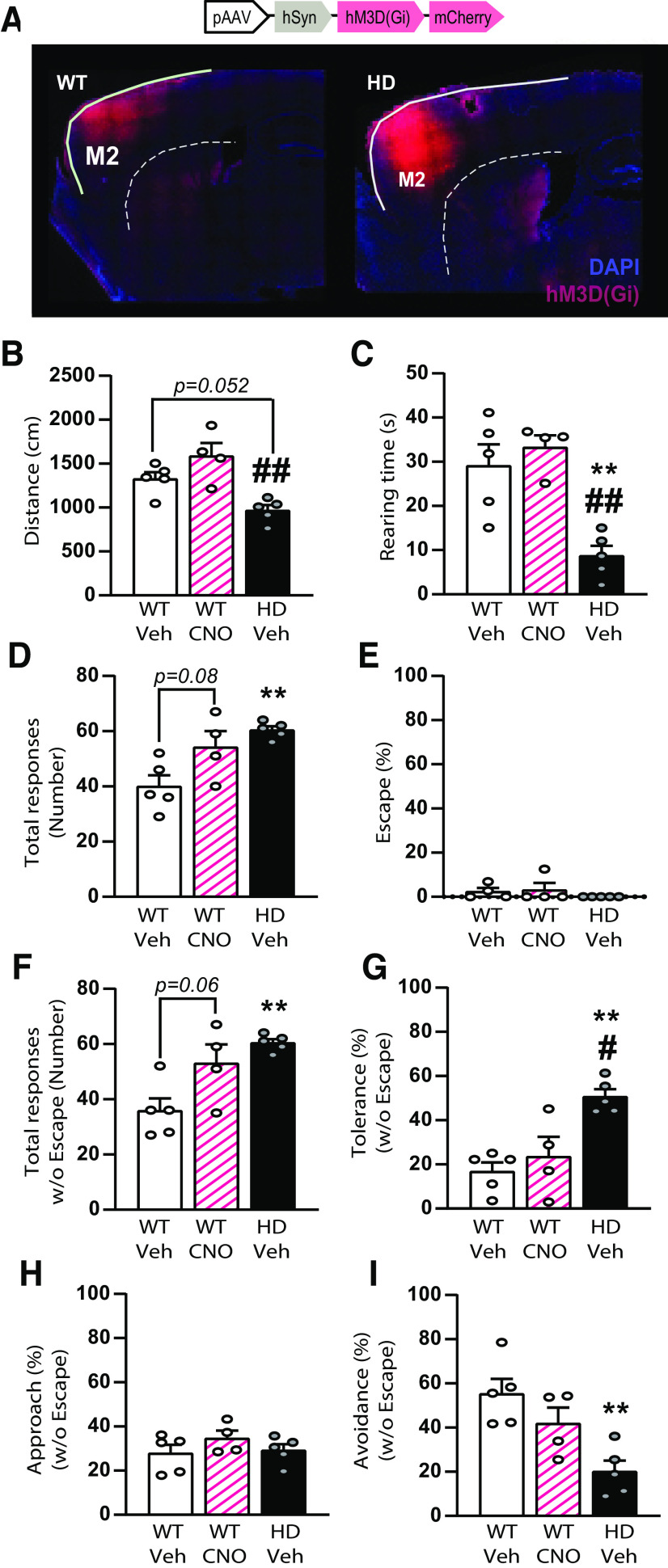
Chemogenetic inhibition of M2 cortical neurons shifts behavioral responses to the robo-beetle in WT mice toward an HD phenotype in the BMT. ***A***, Representative fluorescent image showing M2 cortical neurons expressing AAV5-syn-hdM3D(gi)-mCherry chemogenetic viral constructs (red) and DAPI (blue). ***B***, ***C***, During the habituation phase (***B***) distance traveled and (***C***) rearing time were measured. ***D-I***, During the test phase, (***D***) total responses to the robo-beetle were measured. ***E***, Percentages of escape responses. ***F***, Total responses to the robo-beetle excluding escape. ***G***, Percentage of tolerance, (***H***) approach and (***I***) avoidance responses from total responses excluding the escape response. Each point represents data from an individual mouse. One-way ANOVA followed by Bonferroni *post hoc* analysis was performed. Data are mean ± SEM (WT-Veh *n* = 5, WT-CNO *n* = 4, and HD *n* = 5 mice; 20-week-old mice). *p < 0.05, **p < 0.01, versus WT-Veh. ^#^*p* < 0.05, ^##^*p* < 0.01, versus WT-CNO.

To further understand whether the lack of M2 cortex activity is responsible for the HD phenotype in the BMT, we examined whether acute inhibition of M2 cortical neurons in WT mice could mimic HD responses to a lower visual input. We bilaterally injected mice with AAV5-hSyn-hM3D(Gi)-mCherry in M2 cortex to express the Gi-protein-coupled receptor hM3D and compared the behavioral performance in the BMT ∼40 min after an intraperitoneal injection of CNO (1 mg/kg) or saline (Veh) (Fig. 10).

During the habituation phase, both locomotion and exploratory behavior remain similar between WT vehicle or CNO injected mice, while was reduced in HD mice (Fig. 10*B*,*C*). One-way ANOVA revealed significant group effect in both locomotion (*F*_(2,11)_ = 10.6, *p* = 0.03) and exploratory behavior (*F*_(2,11)_ = 13.5, *p* = 0.001), with only a significant reduction in both parameters in HD mice compared with WT, as revealed by Bonferroni *post hoc* analysis.

During the subsequent testing phase, the behavioral responses toward the robo-beetle were evaluated. The responses toward the robo-beetle in WT CNO-treated mice were shifted toward an HD phenotype (Fig. 10*D–I*). In detail, CNO treatment in WT mice increased total number of responses compared with WT vehicle and got closer to HD vehicle levels (Fig. 10*D*, one-way ANOVA group effect (*F*_(2,11)_ = 7.9, *p* = 0.008)). Escape behavior was mostly absent in this experiment and similar in all groups (Fig. 10*E*, one-way ANOVA group effect (*F*_(2,10)_ = 0.8, *p* = 0.4)). Also, WT CNO increased the total number of responses without escape behavior (Fig. 10*F*, one-way ANOVA group effect (*F*_(2,11)_ = 8.8, *p* = 0.005)), showed a tendency to an increase in tolerance (Fig. 10*G*, one-way ANOVA group effect (*F*_(2,11)_ = 11.5, *p* = 0.002)), similar approach (Fig. 10*H*, one-way ANOVA group effect (*F*_(2,11)_ = 1.0, *p* = 0.4)) and reduced avoidance responses (Fig. 10*I*, one-way ANOVA group effect (*F*_(2,11)_ = 8.6, *p* = 0.006)) compared with WT veh and shifted toward the HD phenotype. Bonferroni *post hoc* analysis further confirmed that inhibition of M2 cortical neurons in WT mice induced a partial HD phenotype, with no differences either to WT Veh or HD veh in any of the responses analyzed, except for the tolerance behavior which was still different to HD veh.

In sum, despite the low number of mice used, acute M2 cortex inhibition in WT mice was able to shift behavioral responses toward an HD phenotype, suggesting that neuronal activity in the M2 cortex is key to select the type of responses on unexpected visual stimuli, such as a randomly moving robo-beetle.

## Discussion

Cortical premotor areas are prominently affected in HD many years before the onset of motor symptoms. These alterations have been widely correlated to cortico-striatal disconnection and associated to motor deficits, which are severely manifested in HD. Here, we thoroughly mapped M2 cortex connectivity in HD mice and described prominent structural and functional defects, particularly in the M2 cortex projection to the SC. Moreover, our data reveal profound alterations in SC-dependent behavioral functions in HD mice, which appear before motor alterations. Moreover, we showed that M2 cortex activity is engaged in the presence of a visual stimuli in WT but not in HD mice, and that acute M2 cortex inhibition using DREADDS promotes an HD-like phenotype in the BMT in WT mice, suggesting that aberrant activation of the M2 cortex might contribute to the observed behavioral alterations in HD mice.

M2 cortex pyramidal neurons send projections to many cortical and subcortical brain nuclei, including the SC, as previously described (Zhang et al., 2016; Economo et al., 2018; Gerfen et al., 2018; Lin et al., 2018). Notably, fMRI shows sever functional connectivity deficits between the M2 cortex and several cortices, hippocampus, thalamus, PAG, and SC in symptomatic HD mice, which differs from the neuroanatomical projections. Because functional connectivity is defined as the temporal correlation between spatially remote neurophysiological events (Fingelkurts et al., 2005), the strong correlation of activity between M2 cortex and hippocampus, thalamus, and PAG might be indirect though additional bran circuits. Nevertheless, the M2 cortex represents one of the major inputs to the SC. Further, deficits in functional connectivity are accompanied by structural M2 cortex–SC circuit alterations, seen by a decrease in DWI metrics. Together, we demonstrate that long-range pyramidal tract neurons from M2 cortex to SC are severely affected both structurally and functional, which contrasts with the idea that intratelencephalic neurons are more affected in HD (Gatto et al., 2015). Moreover, SC structure seems preserved in HD conditions, as shown by DWI measurements in the SC and electrophysiological responses if M2 axons in the SC using MEA, suggesting that alterations in M2 cortex–SC functions are related to M2 cortex inputs rather than deficits in the SC structure.

Consistently, alterations in M2 cortex function might be a common underlying mechanism in diverse HD symptoms. M2 cortex activation has been involved in many naturalistic behaviors, such as rearing, grasping, eating, grooming, etc., as seen by calcium imaging experiments using miniature microscopes (Tombaz et al., 2020) and motor learning processes, seen by two-photon imaging of Arc-GFP expression (Cao et al., 2015). All these behaviors are well known to be altered in HD mouse models (Pépin et al., 2016; Fernández-García et al., 2020). In this line, we demonstrated that aberrant M2 cortex activity contributes to the SC-related behaviors in HD, such as the BMT, associated to visual perception of a thread and dependent on SC function (Comoli et al., 2012; Almada et al., 2018). In detail, HD mice showed reduced escape and avoidance responses, while increased tolerance responses to the robo-beetle. Moreover, M2 cortex activity was engaged in the presence of the robo-beetle in WT but not HD mice, and M2 cortex inhibition using DREADDs mildly shifted WT mice responses toward an HD phenotype in the BMT. Although the low number of mice used in the latter experiment could underestimate the effects of chemogenetic stimulation, our data suggests that M2 cortex activity contributes to the selection of responses on visual stimuli; however, additional SC circuits may also contribute to the HD phenotype.

In this regard, M2 cortex displays the highest functional connectivity with the Cg, which in turn is the main cortical afferent to the medial portion of the SC (mSC) (Savage et al., 2017). Information flows from M2 cortex and Cg to the SC in functionally segregated loops and then goes back to basal ganglia circuitry through distinct thalamic targets to modulate action selection (McHaffie et al., 2005). In detail, M2 cortex–dlSC pathway function is related to approach and appetitive stimuli normally found in the lower visual field (Dean et al., 1986; Sahibzada et al., 1986), while Cg–mSC function is more related to escape behaviors and movements in the upper visual field (e.g., predators) (Comoli et al., 2012; Savage et al., 2017). Surprisingly, we found that the number of escape responses in WT mice were very variable among different mice batches, possibly related to differential anxiety levels between them (Heinz et al., 2017), while HD mice did few or no escape responses in any of them. This significant difference observed in evoked escape responses between genotypes suggest that the Cg cortex–mSC and/or the SC–PAG pathways may be also affected in HD pathology. In addition, M2 cortex activity was engaged in all WT mice on the presence of the robo-beetle, regardless of the presence of escape responses, supporting the idea that escape responses are not dependent on M2 cortex activity. Consequently, the presence of alterations in all the diverse behavioral responses in HD mice suggests that inputs to both the medial and lateral parts of the SC might be affected. Accordingly, Cg is known to be altered in HD patients at early stages (Hobbs et al., 2011), and cell loss in the motor and Cg correlates with symptomatology in HD (Thu et al., 2010). Therefore, we hypothesize that alterations in Cg–SC might also have a role in HD.

In parallel, M2 cortex-PAG circuit is also functionally altered in HD mice, as shown by the fMRI data. The PAG is one of the major outputs of the SC and is responsible for defining the threshold to compute escape responses (Evans et al., 2018; Branco and Redgrave, 2020), which were almost absent in HD conditions. Interestingly, increased GABAergic tone from the ventral lateral geniculate nucleus (Fratzl et al., 2021) or the substantia nigra reticulata (Almada et al., 2018) reduces escape responses in the BMT, suggesting that increased GABAergic activity in the SC could lead to the altered behavioral responses in HD mice. Further, SC-PAG circuit is shaped by multimodal sensory information and learning experiences, which are constantly updated throughout the life of the animal (Evans et al., 2019). Because M2 cortex sends only few direct projections to the PAG (Savage et al., 2017), we hypothesize that M2 cortex might modulate PAG function indirectly through the modulation of SC activity, or alternatively, by additional inputs to the SC and PAG, such as the Cg.

The SC is involved in the control of orienting gaze shifts, also known as saccadic movements (Sparks, 1986; Munoz and Guitton, 1991). Therefore, the observed alterations in SC functions in HD mice are consistent with the abnormal control of saccadic eye movements present in HD patients from presymptomatic stages (Patel et al., 2012; Srivastava et al., 2018). Indeed, oculomotor functions and locomotion share many underlying neuronal circuits (Srivastava et al., 2018) and, in several movement disorders, oculomotor deficits precede motor symptoms (Termsarasab et al., 2015). Whereas direct alterations of SC function in HD patients have not been explored, studies in Parkinson disease (PD) show that responses to luminescence (measured by fMRI) in the SC are abnormal in *de novo* patients compared with controls (Moro et al., 2020). These alterations in the SC are linked to deficits in the coordination of action and perception (Pretegiani et al., 2019). Notably, a recent study with PD patients show that repetitive transcranial magnetic stimulation in the motor cortex improved anti-saccade success rate and postural instability gait difficulty (Okada et al., 2021). In this line, optogenetic stimulation of the M2 cortex reverts motor dysfunction in a mouse model of PD (Magno et al., 2019). Thus, further understanding on the complex circuitry between cortical, visual, and motor domains could help to design new treatment opportunities based on circuit restoration in movement disorders.

In conclusion, our data provide compelling evidence of the involvement of M2 cortex circuitry alterations to HD pathophysiology, beyond basal ganglia. Particularly, we highlight the alterations in M2 cortex functional connectivity and particularly the lack of M2 cortex engagement as contributor to the SC-dependent symptoms observed in HD mice. Unraveling the myriad of circuit alterations and functions from M2 cortex other than cortico-striatal provides valuable insights to understand HD symptoms, which might be also relevant for other movement disorders, such as PD. Moreover, further understanding and targeting SC circuitry may additionally provide novel therapeutic opportunities aiming to delay the onset and severity of symptoms in movement disorders.
